# Complex patterns arise through spontaneous symmetry breaking in dense homogeneous networks of neural oscillators

**DOI:** 10.1038/srep22074

**Published:** 2016-02-26

**Authors:** Rajeev Singh, Shakti N. Menon, Sitabhra Sinha

**Affiliations:** 1 The Institute of Mathematical Sciences, CIT Campus, Taramani, Chennai 600113, India; 2 National Institute of Advanced Studies, Indian Institute of Science Campus, Bangalore 560012, India

## Abstract

There has been much interest in understanding collective dynamics in networks of brain regions due to their role in behavior and cognitive function. Here we show that a simple, homogeneous system of densely connected oscillators, representing the aggregate activity of local brain regions, can exhibit a rich variety of dynamical patterns emerging via spontaneous breaking of permutation or translational symmetries. Upon removing just a few connections, we observe a striking departure from the mean-field limit in terms of the collective dynamics, which implies that the sparsity of these networks may have very important consequences. Our results suggest that the origins of some of the complicated activity patterns seen in the brain may be understood even with simple connection topologies.

Collective dynamics of coupled oscillators, in particular, synchronization^1^, is integral to many natural phenomena^2^ and is especially important for several biological processes^3^,^4^,^5^, such as brain function^6^,^7^,^8^,^9^,^10^. While very large-scale synchronization of neuronal activity is considered pathological, as in epilepsy^11^, the brain is capable of exhibiting a variety of complex spatiotemporal excitation patterns that may play a crucial role in information processing^12^. Understanding the dynamics of these patterns at the scale of the entire brain (imaged using techniques such as fMRI) is of fundamental importance^13^. As detailed simulation of each individual neuron in the brain is computationally expensive,^14^,^15^ when studying the dynamics of the entire system it is useful to focus on the network of interactions between brain regions. It has also been explicitly shown that the collective response of a large number of connected excitatory and inhibitory neurons, which constitute such regions, can be much simpler than the dynamics of individual neurons^16^. Indeed, each region can be described using phenomenological models in terms of a few aggregate variables^17^.

Using anatomical and physiological data collected over several decades, the networks of brain regions for different animals have been reconstructed^18^,^19^,^20^, where the individual nodes correspond to large assemblies (10^3^–10^6^) of neurons^21^,^22^. The connectivity *C* (i.e., fraction of realized links) of these networks (*C* ~ 10^−1^) is significantly higher than that among neurons (*C* ~ 10^−6^)^23^,^24^. A schematic representation of a network of the Macaque brain regions (adapted from a recent study^20^; see Methods for more details) is shown in Fig. 1(a). The collective activity of such networks can result in complicated nodal dynamics, including temporal oscillations at several scales that are known to be functionally relevant^10^,^25^,^26^. Each of these nodes can be described using neural field models of localized neuronal population activity, which can have varying mathematical complexity and biological realism^27^,^28^,^29^. Here, we use the well-known and pioneering model proposed by Wilson and Cowan (WC)^30^,^31^ to describe the activity of each brain region.

The complex collective dynamics obtained using this model for the Macaque network, shown in Fig. 1(b), are reminiscent of experimentally recorded activity of brain regions^10^. The range of behaviors observed in this system at different connection strengths [Fig. 1(c,d)], can arise from an interplay of several factors, which makes their analysis difficult. A possible approach to understand the genesis of these patterns is to focus on the dynamics of nodes interacting in the simplified setting of a homogeneous, globally coupled system, which is an idealization of the densely connected network.

In this article we show that this simple system exhibits an unexpectedly rich variety of complex phenomena, despite lacking the detailed topological features of brain networks [e.g., Fig. 1(a)], such as heterogeneity in degree (number of connections per node) and modular organization. In particular, we show the existence of novel collective states, including those characterized by oscillator clusters, where each cluster is distinguished by its amplitude or frequency. The occurrence of such clusters is surprising as each node is identical in terms of both intrinsic dynamics and connectivity, indicating that the homogeneous system of oscillators undergoes *spontaneous symmetry breaking*. In addition we observe patterns where the time-series for all oscillators are identical except for a non-zero phase difference between groups of exactly synchronized elements that we refer to as “phase clusters”. On removing a few links from the network while preserving the structural symmetry of connections we observe even more striking situations such as the appearance of many (>2) clusters having different amplitudes. Moreover, oscillator death (i.e., termination of oscillations upon coupling), which is seen over a substantial region of parameter space in the fully connected system, occurs in a drastically reduced region for such marginally sparse networks. As the behavior of a large, densely connected system is effectively identical to that of the corresponding mean-field model, it is remarkable that the dynamical properties of the system considered here are radically altered in response to extremely minor deviations from the fully connected situation.

## Results

We first examine the collective dynamics of a pair of coupled oscillators (*N* = 2) as a function of the interaction strength *w* between them. Figure 2(a,b) show that while exact synchronization (ES) of oscillator dynamics occurs at weak coupling (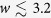), a state of anti-phase synchronization (APS) is observed at higher values of *w* (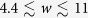). For intermediate *w*, the co-existence of the dominant frequencies corresponding to ES and APS states [Fig. 2(c)] indicates that the quasi-periodic (QP) behavior observed in this regime can be interpreted as arising through competition between the mechanisms responsible for the above two states. At *w* ~ 11, the system undergoes spontaneous symmetry-breaking, eventually giving rise to inhomogeneous in-phase synchronization (IIS), characterized by different phase space projections and distinct amplitudes for the time-series of each oscillator [Fig. 2(a,b), last panel]. The nature of the transition from APS to IIS is made explicit in Fig. 2(d) [top panel], where the excitatory components (*u*_1,2_) of the fixed points of the system, obtained using numerical root finding, are shown over a range of *w*. At *w* ≈ 10.943, a pair of heterogeneous unstable solutions related by permutation symmetry, corresponding to an inhomogeneous steady-state (ISS), emerge from a homogeneous unstable solution, beyond which all three solutions coexist. Thus, spontaneous symmetry breaking appears to arise in the system through a subcritical pitchfork bifurcation, with the number of positive eigenvalues corresponding to the homogeneous solution decreasing by unity (not shown). The ISS is stable over a very small range, 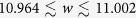, as seen from their corresponding eigenvalues in Fig. 2(d) [lower panel]. Note that stability is lost on either end of this interval through supercritical Hopf bifurcations (see Supplemental Material). For 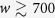, both oscillators converge to the inactive state *u*_*i*_ = *v*_*i*_ = 0 ∀*i*, corresponding to amplitude death (AD, not shown).

Increasing the number of oscillators, we observe that while the patterns seen for a pair of coupled oscillators, namely ES, QP, ISS, IIS, APS and AD persist (first four shown in Fig. 3(a,b) for *N* = 20) qualitatively different states also emerge as shown in the phase diagram in Fig. 3(c). As mentioned earlier, a new class of patterns characterized by the existence of phase clusters appears. The most robust of these, referred to as gradient synchronization (GS), has *n*_*cl*_ ~ *N* clusters with distinct phases. Note that APS, which for *N* > 2 has a very small basin of attraction, is a phase cluster state for which *n*_*cl*_ = 2. Another new pattern comprises two oscillator clusters, each characterized by a unique frequency [Fig. 3(d)]. This constitutes a dramatic instance of spontaneous breaking of permutation symmetry, as the oscillators are intrinsically indistinguishable for this completely homogeneous system. Thus, the appearance of multiple frequencies in a dynamical network need not imply heterogeneity in connectivity or node properties. A third new pattern is a homogeneous steady state referred to as oscillator death (OD), in which the individual nodes have the same time-invariant, non-zero activity. This dynamical state appears over a large region in (*w*,*N*)-space as seen in the phase diagram, Fig. 3(c). To identify and segregate the regimes in this diagram, we use several order parameters (see Methods for details, and the table in the Supplemental Material for a summary). A system with a small number of oscillators can show multistability, i.e., different initial conditions may converge to distinct dynamical regimes for identical parameter values, close to the boundary between two regimes. Thus, we identify the regime to which a point in the (*w*, *N*)-space belongs as the one to which a majority (>50%) of the initial conditions converge. However, as *N* increases the regions showing such multistability shrinks, as shown by the finite-size scaling of the ES-QP boundary [Fig. 3(e)] that decreases as 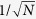.

**Figure 1:**
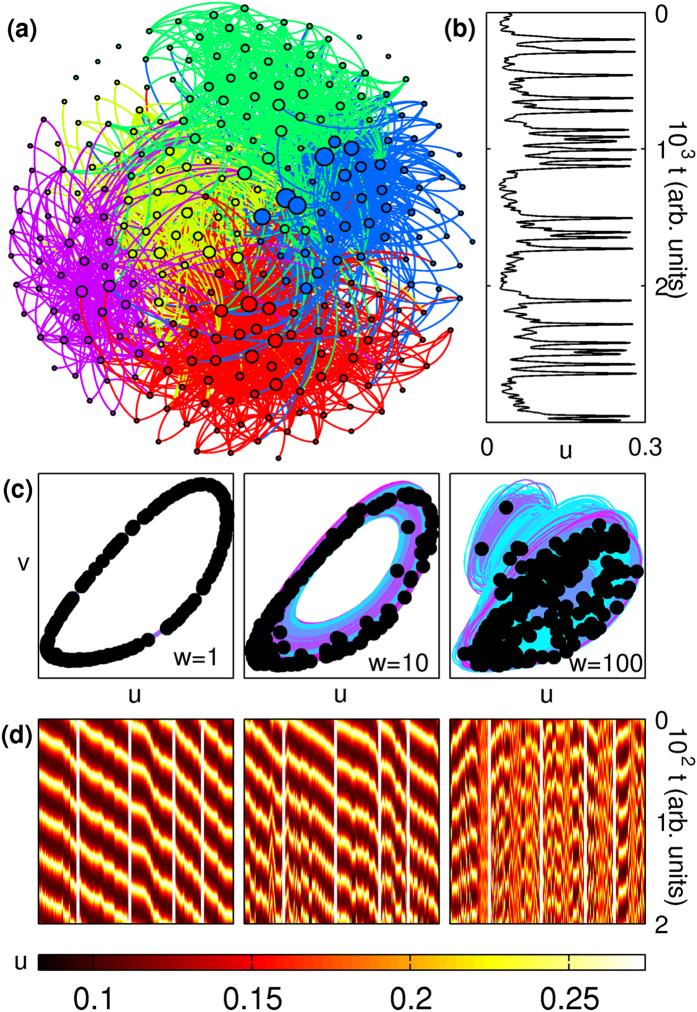
(**a**) The directed network of connections between regions of the Macaque brain, adapted from a recent study^20^ (see Methods for more details). The size of each node is proportional to its total degree and the colors distinguish the modules (characterized by significantly higher intra-connection density and obtained using a partitioning algorithm^39^). The color of each link corresponds to that of the source node. We have used the Fruchterman-Reingold algorithm, as implemented in Gephi^40^, for placing the nodes of the network in a two-dimensional plane so that all links are of approximately equal length and cross each other minimally. This force-directed placement of nodes seems to clearly segregate the modules obtained with the partitioning algorithm used here. (**b**) Time series of the excitatory component of a typical node in this network with coupling strength *w* = 500, where each node is modeled as a Wilson-Cowan oscillator. (**c**) Phase space projections of the oscillators, obtained for different coupling strengths, where the filled circles represent the location of each oscillator at a time instant. The panels here are scaled individually for better visualization. (**d**) Time-series of the excitatory component *u*, for the corresponding values of *w* used in the panels directly above. The nodes *i* are arranged according to their modules (demarcated by white lines).

**Figure 2:**
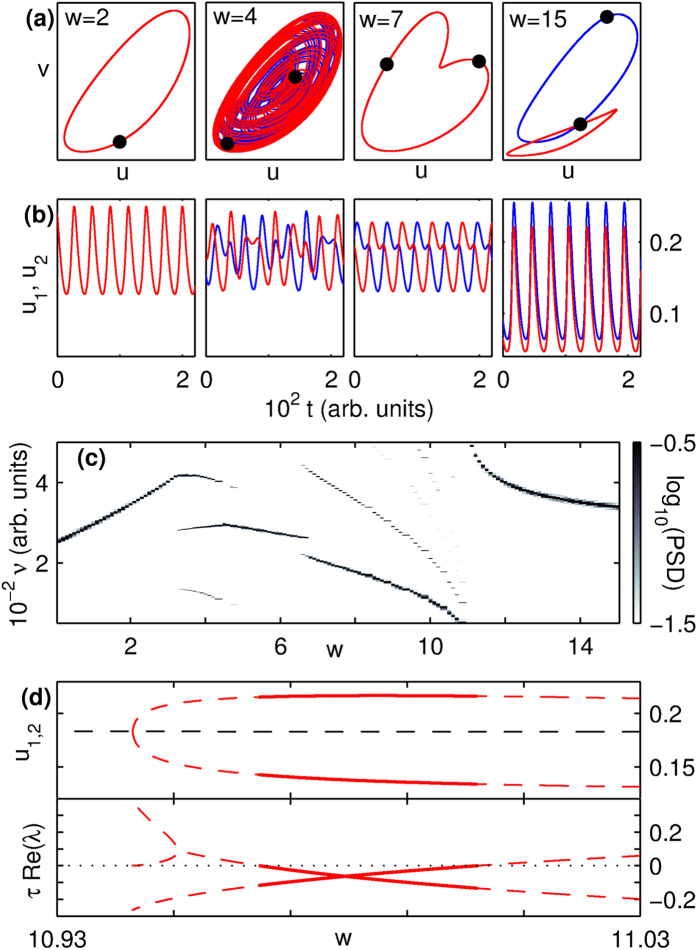
Collective dynamics of a system of two coupled WC oscillators. (**a**) Phase space projections of the trajectories and (**b**) time-series for each oscillator showing exact synchronization (ES, for coupling *w* = 2), quasiperiodicity (QP, *w* = 4), anti-phase synchronization (APS, *w* = 7) and inhomogeneous in-phase synchronization (IIS, *w* = 15). The filled circles represent the location of each oscillator in phase space at a time instant. The panels in (**a**) are scaled individually for better visualization. (**c**) Power-spectral density (PSD) of the time-series for the *u* component of each oscillator, revealing the dominant frequencies as a function of *w*. (**d**) Excitatory components of the fixed points of the system (upper panel) and the real parts of the eigenvalues corresponding to the heterogeneous fixed points (lower panel) showing the transitions between APS and IIS regimes (see Supplemental Material), where *τ* = *τ*_*u*,*v*_ = 8. Solid (broken) lines represent stable (unstable) solutions. The horizontal broken line (upper panel) represents the unstable homogeneous solution.

**Figure 3:**
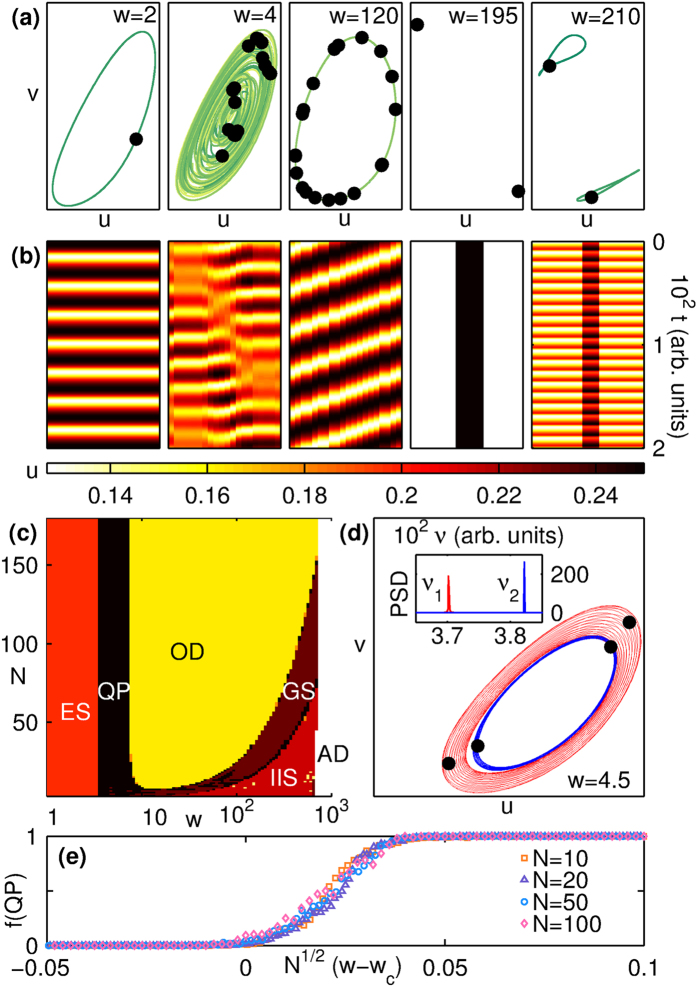
Collective dynamics of *N* densely connected WC oscillators. (**a**) Phase space projections of the trajectories and (**b**) time-series for *N* = 20 globally coupled oscillators, showing exact synchronization (ES, *w* = 2), quasiperiodicity (QP, *w* = 4), gradient synchronization (GS, *w* = 120), inhomogeneous steady-state (ISS, *w* = 195) and inhomogeneous in-phase synchronization (IIS, *w* = 210) (see Supplemental Material). The panels in (**a**) are scaled individually for better visualization. (**c**) Phase diagram for *N* WC oscillators globally coupled with strength *w*, indicating areas where the majority (>50%) of initial conditions result in ES, QP, GS, IIS, oscillator death (OD) and amplitude death (AD). Note that the *w*-axis is logarithmic. (**d**) Phase space projections of the different oscillators (*N* = 20) for *w* = 4.5, which form two clusters with frequencies *ν*_1_ and *ν*_2_, indicated by the power-spectral density (PSD, inset). (**e**) Finite-size scaling of the fraction of initial states that converge to QP, shown as a function of *w* at the boundary between the ES and QP regimes, for different system sizes *N*.

**Figure 4:**
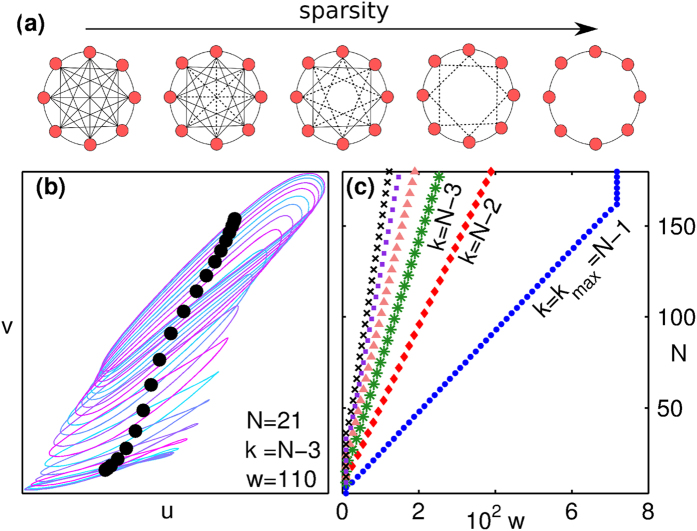
(**a**) Schematic diagram indicating the procedure for degree reduction to systematically make a homogeneous network increasingly sparse, while preserving as many of the existing symmetries as possible. (**b**) When the degree *k*, i.e., the number of links per node, deviates slightly from the globally coupled case (*k*_max_ = *N* − 1) to *N* − 3, the trajectories of the IIS state split into many (~*N*) distinct projections (*N* = 21, *w* = 110). (**c**) The extent of the OD region in Fig. 3(c) is seen to shrink rapidly with the number of removed links.

As a first step towards extending the results seen for the globally coupled system to brain networks of the type shown in Fig. 1(a), we have investigated the consequences of gradually decreasing the connection density (see Methods for details). In addition to preserving degree homogeneity, our method ensures that every node has the same neighborhood structure [Fig. 4(a)]. As we deviate from the global coupling limit, we observe patterns similar to those shown in Fig. 3(a–d), although the precise form of the attractors may differ and it is now the translational symmetry that is being spontaneously broken. For example, as seen in Fig. 4(b), a reduction of just 2 links per node causes the trajectory in the IIS state to split into many more (~*N*) projections than seen for the fully connected case (~2). Also, while the phase diagram of the system remains qualitatively unchanged when the degree is decreased from *k*_max_ = *N* − 1, there is a dramatic quantitative reduction in the area corresponding to OD [Fig. 4(c)] even with the reduction of one link per node. This is surprising, as one would expect that a marginal deviation from the global coupling limit in large systems will not result in a perceptible change from the mean-field behavior.

As it is well-known that the incorporation of time delays can qualitatively change the observed patterns in dynamical systems^32^ (including neural oscillators^33^,^34^), and can impact their stability^35^, we have also simulated a system of globally coupled oscillators incorporating a delay in each of the links (see Methods for more details). We observe that while the precise nature of the synchronization pattern changes on the introduction of delay, the patterns observed fall under the same qualitative categories as those described above (see Supplemental Material). Intriguingly, it would appear that a small amount of delay tends to suppress the complexity of collective dynamics by enhancing the likelihood of exact synchronization being observed, even at strong coupling strengths. However, complicated patterns re-emerge as the delays get longer.

## Discussion

An important implication of this study follows from our demonstration that systems with simple connection topology are capable of exhibiting very rich dynamical behavior. In particular, many of the patterns seen in our simulations of the network of Macaque brain regions (Fig. 1) resemble those observed using much simpler connectivity schemes (Fig. 3). Hence, patterns seen in complex systems that are often attributed to their non-trivial connection structure, may in fact be independent of the details of the network architecture.

Our result that weakening connections between nodes of a network can increase coherence in collective activity (viz., observation of ES at low *w*) suggests an intriguing relation between two recent experimental findings: (i) anaesthetic-induced loss of consciousness occurs through the progressive disruption of communication between brain areas^36^ and (ii) functional connectivity networks reconstructed from EEG data become increasingly dense with the development of fatigue in sleep-deprived subjects^37^,^38^. The latter study finds that the onset of sleep is accompanied by an increase in the degree of synchronization between brain areas, while the former result implies that the interaction strengths between these areas will concurrently get weaker. Although it may appear counter-intuitive that decreased coupling strength would result in increased synchronization, our findings illustrate that these results are not incompatible.

With the availability of high-resolution data and increased computational power, it is now possible to model brain networks incorporating a higher level of realistic detail^14^. Nevertheless even simple phenomenological models on coarse-grained brain-region networks may provide insights into mesoscale phenomena, such as those observed in fMRI and EEG experiments. Our findings provide a baseline for future investigations of the specific role of the detailed aspects (such as degree heterogeneity or modular architecture) of brain networks on their collective dynamics. Indeed, a preliminary study into the role of node heterogeneity (in terms of the application of external stimulus) indicates that a rich variety of novel effects, such as coupling-induced oscillations, can be observed. To conclude, we have shown that the collective dynamics of a homogeneous system of oscillators, motivated by mesoscopic descriptions of brain activity, exhibits spontaneous symmetry breaking that gives rise to several novel patterns. Despite preserving the structural symmetry of connections, a marginal increase in the network sparsity, corresponding to an extremely small deviation from the mean-field, unexpectedly changes the robustness of certain patterns. Our results suggest that some of the complicated activity patterns seen in the brain can be explained even with simple connection topologies.

## Methods

### Nodal dynamics

The model we consider comprises a network of *N* oscillators, each described by the WC model whose dynamics results from interactions between an excitatory and an inhibitory neuronal subpopulation. The average activity of each node *i* (*u*_*i*_, *v*_*i*_) evolves as:


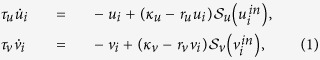


where 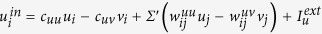 and 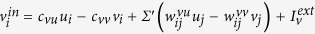 represent the total input to the two subpopulations respectively. The time constants and external stimuli for the subpopulations are indicated by *τ*_*u*,*v*_ and 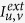 respectively, while *c*_*μν*_ (*μ*, *ν* = *u*, *v*) corresponds to the strength of interactions within and between the subpopulations of a node. The interaction strengths are represented by the weight matrices 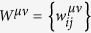 and the summation Σ′ is over all network neighbors (for a globally coupled system, 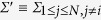). The function 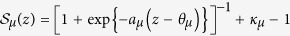 has a sigmoidal dependence on *z*, with *κ*_*μ*_ = 1 − [1 + exp(*a*_*μ*_*θ*_*μ*_)]^−1^. The parameter values have been chosen such that each isolated node (*W*^*μν*^ = 0) is in the oscillatory regime, viz., *a*_*u*_ = 1.3, *θ*_*u*_ = 4, *a*_*v*_ = 2, *θ*_*v*_ = 3.7, *c*_*uu*_ = 16, *c*_*uv*_ = 12, *c*_*vu*_ = 15, *c*_*vv*_ = 3, *r*_*u*_ = 1, *r*_*v*_ = 1, *τ*_*u*_ = 8, *τ*_*v*_ = 8, 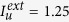 and 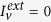. For the homogeneous systems considered here the links all have same strength, i.e., 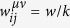 (*μ*, *ν* = *u*, *v*) and *i*(≠ *j*) = 1, …, *N*; 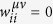, where *k* is the degree of a node. In order to investigate the effect of incorporating a delay *d* in the coupling term, we modify the summation term in 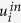 and 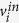 to 
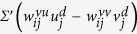
, where 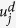 and 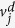 are the activation and inhibition variables of node *j* at a time instant *t* − *d* prior to the current time *t*.

The dynamical system (Eq. 1) is numerically solved using an adaptive-step Runge-Kutta integration scheme for different system sizes (*N*) and coupling strengths (*w*). Linear stability analysis is used to determine the stability of some of the patterns and identify the associated bifurcations. The behavior of the system for each set (*w*, *N*) is analyzed over many (~100) randomly chosen initial conditions. We have explicitly verified that our results are robust with respect to small variations in the parameters.

### Network topology

We use the model outlined above to describe the excitatory-inhibitory dynamics at each node of a network. The resulting synchronization patterns are investigated for three distinct network topologies: (i) Globally coupled networks of *N* nodes, in which each node is (bi-directionally) connected to every other node in the network, excluding itself. (ii) Sparse, symmetric networks, characterized by nodes with identical connection topologies. Sparsity is systematically introduced through a degree reduction that preserves as many of the existing symmetries as possible. To do this, we arrange the nodes on a circle and sequentially remove connections between nodes placed furthest apart. (iii) A realistic network comprising 266 nodes describing the anatomical connectivity between different regions of the Macaque brain. The network we have used here is a reduced version of that presented in a recent study^20^ in that only those brain regions that cannot be further hierarchically subdivided have been considered. This is to avoid redundancies that arise from the fact that several of the nodes in the network of ref. 20 are actually anatomical subdivisions of other nodes. Hence, by only considering nodes at the lowest hierarchical level, we avoid the possibility that the network contains multiple links that describe the same anatomical connection. We have identified the community organization of this network using a partitioning algorithm^39^ that segregates it into five modules (comprising 71, 60, 54, 42 and 39 nodes, respectively).

### Order parameters

The different synchronization patterns are classified through the use of order parameters as follows. The mean of the oscillation amplitude, measured as the variance (*σ*^2^) with respect to time of one of the WC variables, *v*, averaged over all the nodes 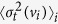, is zero for all the non-oscillating states AD, OD and ISS. These are further distinguished by using the mean and variance with respect to all nodes of the time-averaged *v*, i.e., 〈〈*v*_*i*_〉_*t*_〉_*i*_ (= 0 for AD) and 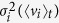 (= 0 for OD and AD). Note that the order parameter 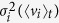 captures aspects of symmetry-breaking, as non-zero values indicate that different oscillators exhibit qualitatively distinct dynamics despite being identical. To distinguish between the oscillating patterns, we consider the mean coherence, measured as 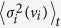, and the total space occupied by all the trajectory projections Δ, as measured by the number of non-zero bins of their histogram in (*u*, *v*)-space. ES is characterized by 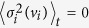 and IIS by 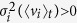. The remaining patterns, GS and QP, are distinguished by Δ being considerably larger for QP. Note that 〈〉_*t*_ and 〈〉_*i*_ represent averaging over time and all nodes, respectively. In practice, different regimes are characterized by thresholds whose specific values do not affect the qualitative nature of the results.

## Additional Information

**How to cite this article**: Singh, R. *et al.* Complex patterns arise through spontaneous symmetry breaking in dense homogeneous networks of neural oscillators. *Sci. Rep.* **6**, 22074; doi: 10.1038/srep22074 (2016).

## Supplementary Material

Supplementary Information

Supplementary Video 1

Supplementary Video 2

Supplementary Video 3

Supplementary Video 4

Supplementary Video 5

Supplementary Video 6

Supplementary Video 7

Supplementary Video 8
