# Supplementary material for: Complex patterns arise through spontaneous symmetry breaking in dense homogeneous networks of neural oscillators
Source: Sci Rep. 2016 Feb 26;6:22074. doi: 10.1038/srep22074 (PMC4768139; doi:10.1038/srep22074)
Supplement: Supplementary Information [file srep22074-s1.pdf]

SUPPLEMENTARY MATERIAL FOR
Complex patterns arise through spontaneous symmetry breaking in dense homogeneous networks of neural oscillators

Rajeev Singh, Shakti N. Menon and Sitabhra Sinha

Contents

1. Fig S1: Bifurcation diagram for a pair of coupled WC oscillators with coupling strength w as the bifurcation parameter, obtained for a set of 20 random initial conditions (i.c.). Red dots represent the maxima of the inhibitory components v for each i.c., while blue dots represent the corresponding minima.

2. Fig S2: Bifurcation diagram for $N = 20$ globally coupled WC oscillators with coupling strength w as the bifurcation parameter, obtained for a set of 20 random initial conditions (i.c.). Red dots represent the maxima of the inhibitory components v for each i.c., while blue dots represent the corresponding minima.

3. Fig S3: Order parameters for identifying different dynamical regimes shown as a function of the coupling strength w for a pair of coupled WC oscillators. The intervals of w where the distinct regimes, viz., ES, QP, APS, IIS and AD, occur are indicated in the figure. Table S1 indicates how the different order parameters are related to these regimes. The order parameter Δ is normalized with respect to the total number of bins (Δ_{tot}) that are used for computing the histogram in (u, v)-space. For an explanation of the order parameters see Methods section.

4. Fig S4: Real parts of the eigenvalues of all the fixed points for a pair of coupled WC oscillators, shown as a function of coupling strength w in the neighborhood of the transition between APS and IIS regimes. The vertical dotted lines indicate the locations where different bifurcations occur in this range of w. Thick lines between the two Hopf bifurcations represent stable solutions. Three of the branches shown correspond to a pair of eigenvalues, as indicated in the figure.

5. Fig S5: Phase plane projections of the trajectories obtained when incorporating delayed coupling in a system of $N = 20$ globally coupled WC oscillators. Results are shown for a range of values of the coupling strength (along the rows), and for different delay times (along the columns).

6. Table S1: Order parameters used for identifying the different dynamical regimes of a homogeneous network of WC oscillators (as explained in the main text).

7. Description of the movies

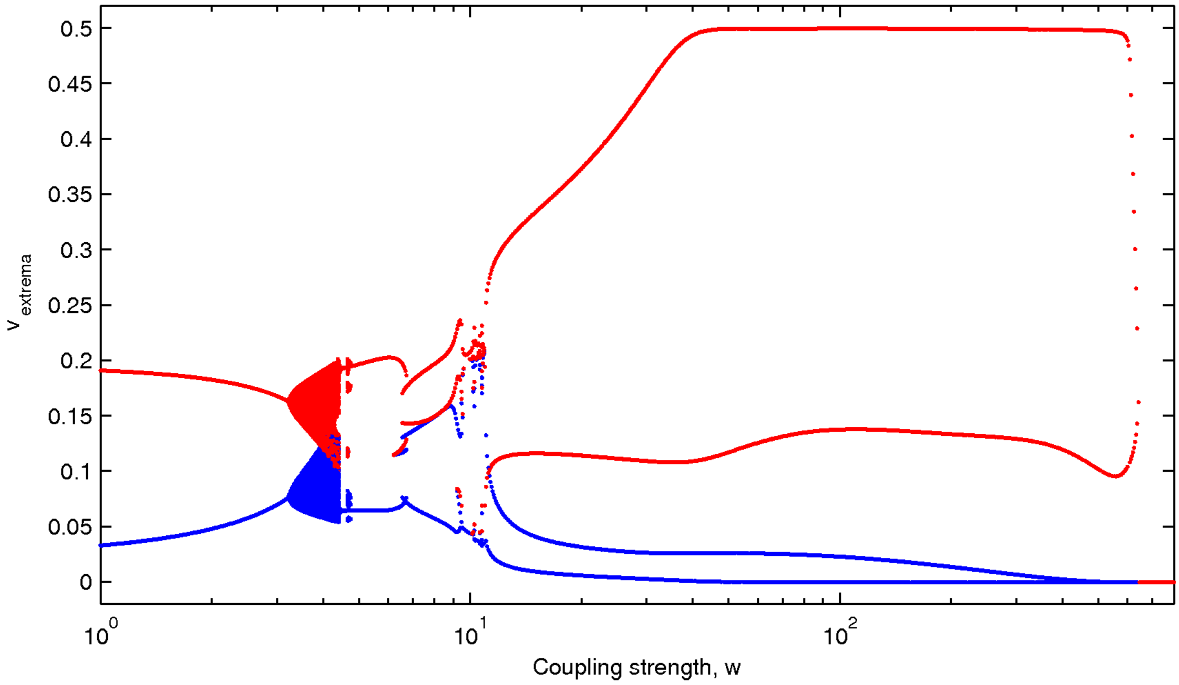

FIG. S1: Bifurcation diagram for a pair of coupled WC oscillators with coupling strength w as the bifurcation parameter, obtained for a set of 20 random initial conditions (i.c.). Red dots represent the maxima of the inhibitory components v for each i.c., while blue dots represent the corresponding minima.

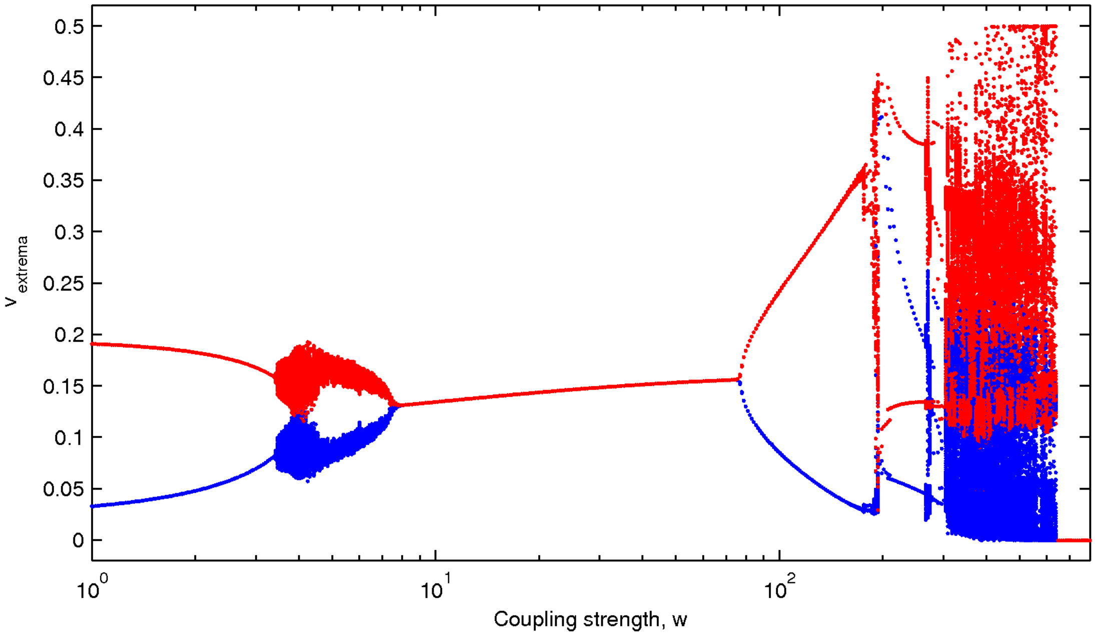

FIG. S2: Bifurcation diagram for $N = 20$ globally coupled WC oscillators with coupling strength w as the bifurcation parameter, obtained for a set of 20 random initial conditions (i.c.). Red dots represent the maxima of the inhibitory components v for each i.c., while blue dots represent the corresponding minima.

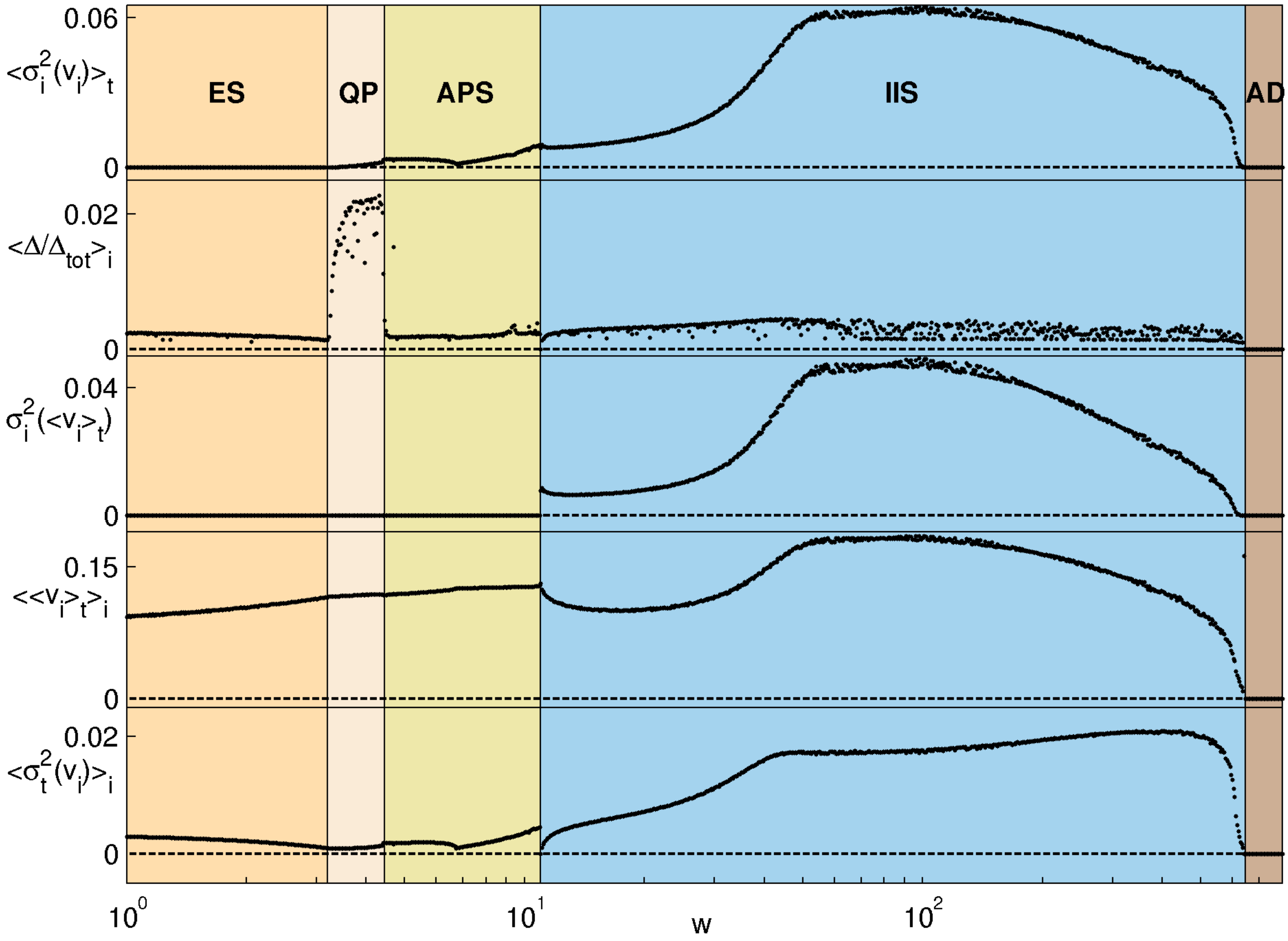

FIG. S3: Order parameters for identifying different dynamical regimes shown as a function of the coupling strength w for a pair of coupled WC oscillators. The intervals of w where the distinct regimes, viz., ES, QP, APS, IIS and AD, occur are indicated in the figure. Table S1 indicates how the different order parameters are related to these regimes. The order parameter Δ is normalized with respect to the total number of bins (Δ_{tot}) that are used for computing the histogram in (u, v)-space. For an explanation of the order parameters see Methods section.

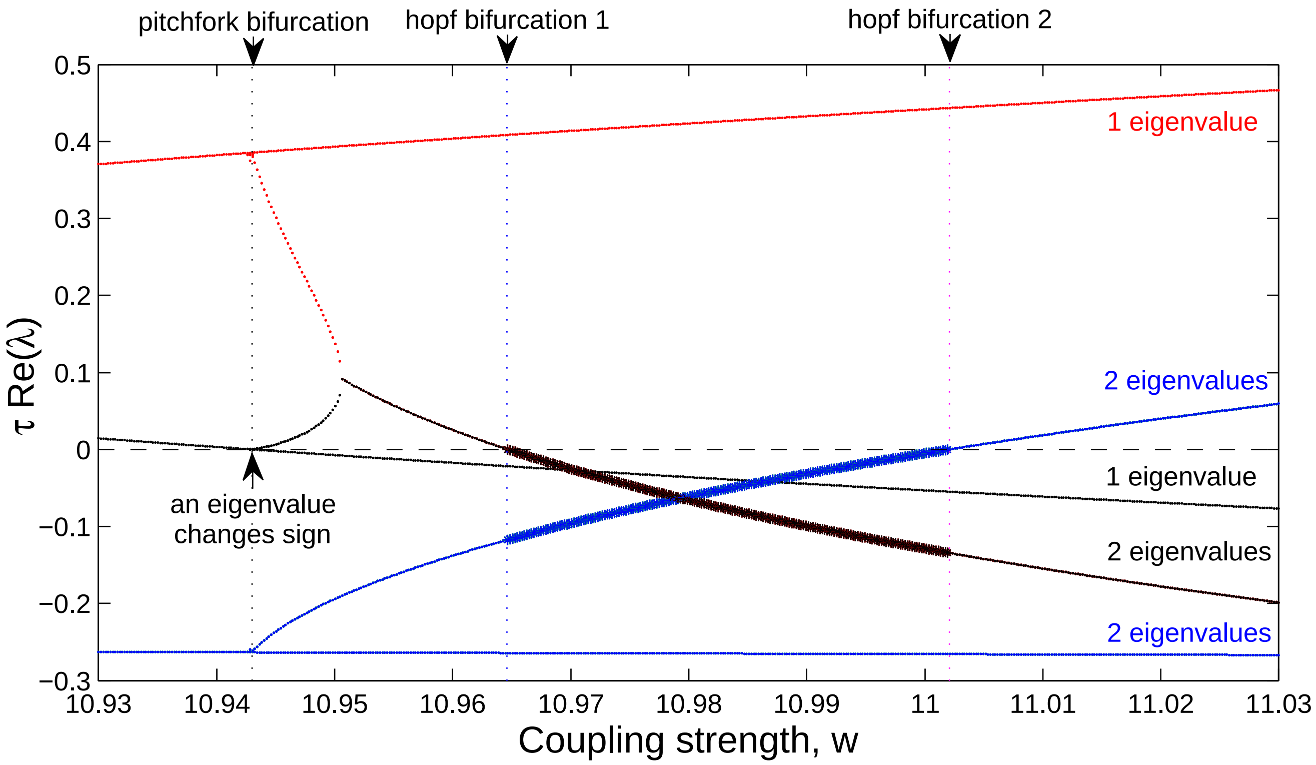

FIG. S4: Real parts of the eigenvalues of all the fixed points for a pair of coupled WC oscillators, shown as a function of coupling strength w in the neighborhood of the transition between APS and IIS regimes. The vertical dotted lines indicate the locations where different bifurcations occur in this range of w. Thick lines between the two Hopf bifurcations represent stable solutions. Three of the branches shown correspond to a pair of eigenvalues, as indicated in the figure. Here $\tau = \tau_{u,v} = 8$.

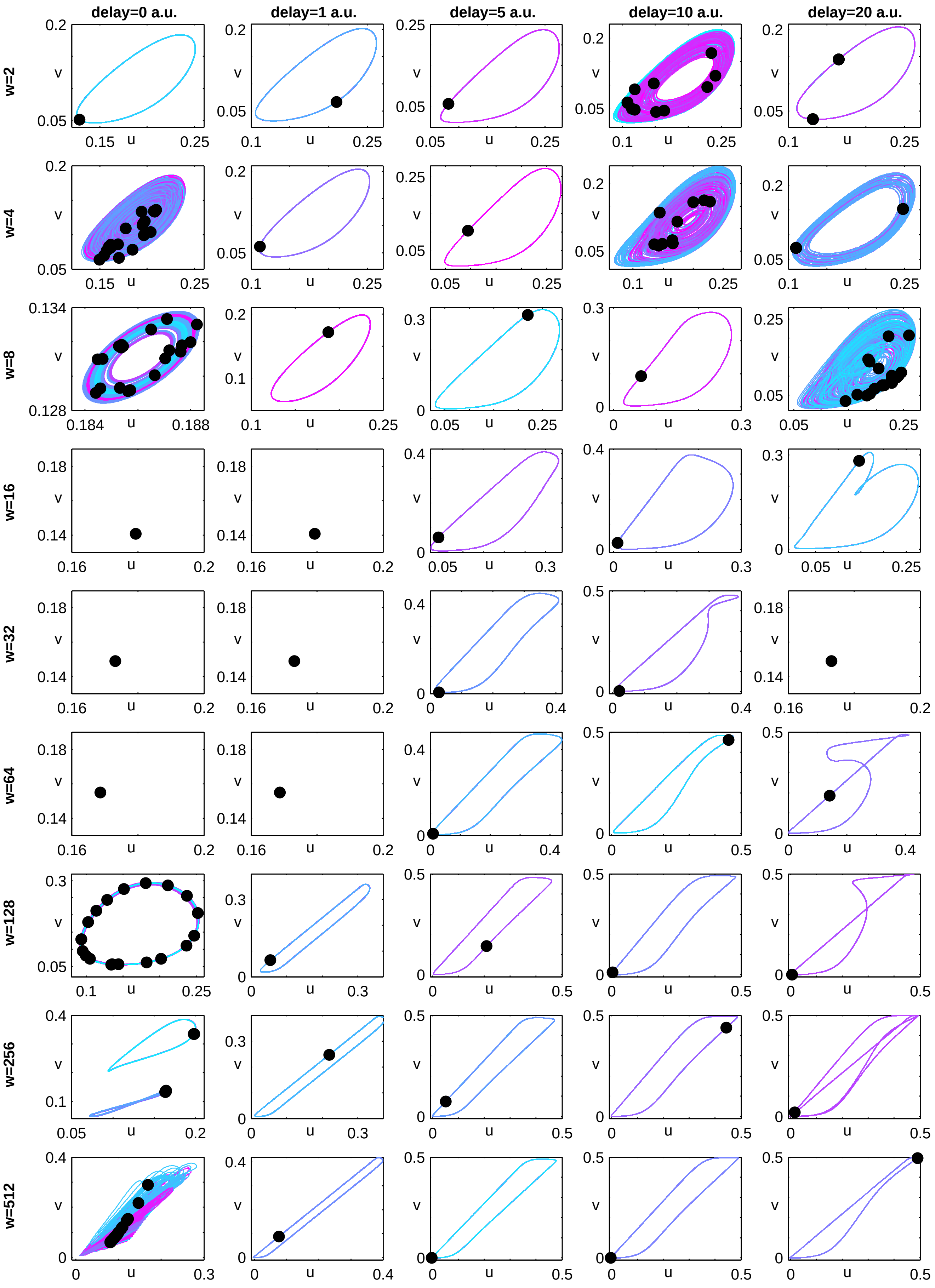

FIG. S5: Phase plane projections of the trajectories obtained when incorporating delayed coupling in a system of $N = 20$ globally coupled WC oscillators. Results are shown for a range of values of the coupling strength (along the rows), and for different delay times (along the columns).

TABLE S1: Order parameters used for identifying the different dynamical regimes of a homogeneous network of WC oscillators (as explained in the main text).

Pattern	$\langle\sigma_t^2(v_i)\rangle_i = 0$	$\langle\langle v_i\rangle_t\rangle_i = 0$	$\sigma_i^2(\langle v_i\rangle_t) = 0$	$\langle\sigma_i^2(v_i)\rangle_t = 0$	$\Delta \gg 0$
AD	✓	✓	✓	✓	
OD	✓		✓	✓	
ISS	✓				
ES			✓	✓	
QP					✓
IIS					
GS			✓		

Description of the movies

The movies are generated from snapshots of the (u, v) phase space projections of N coupled WC oscillators. The black curve in each movie corresponds to the limit cycle of individual oscillators in the absence of coupling. The colored curves represent the long time behavior of each oscillator which asymptotically will converge to the corresponding attractor projections shown in the figures in the main text. In each case, the initial position of each oscillator in (u, v) phase space is chosen randomly from the unperturbed limit cycle. The movies show the time-evolution from this random initial state, including the transient behavior. The state shown by each movie is indicated below:

- `Movie1_N_2__ES.avi`

  Exact synchronization state for $N = 2$ coupled oscillators.

- `Movie2_N_2__QP.avi`

  Quasi-periodic state for $N = 2$ coupled oscillators.

- `Movie3_N_2__APS.avi`

  Anti-phase synchronization state for $N = 2$ coupled oscillators.

- `Movie4_N_2__IIS.avi`

  In-homogeneous in-phase synchronization state for $N = 2$ coupled oscillators.

- `Movie5_global_coupling_N_20__QP.avi`

  Quasi-periodic state for $N = 20$ globally coupled oscillators.

- `Movie6_global_coupling_N_20__GS.avi`

  Gradient synchronization state for $N = 20$ globally coupled oscillators.

- `Movie7_global_coupling_N_20__IIS.avi`

  In-homogeneous in-phase synchronization state for $N = 20$ globally coupled oscillators.

- `Movie8_marginally_sparse_N_21_k_18__IIS.avi`

  In-homogeneous in-phase synchronization state for $N = 21$ coupled oscillators in a marginally sparse network where each oscillator is coupled to $k = 18$ other oscillators.
